# Less Is Better. Avoiding Redundant Measurements in Studies on Wild Birds in Accordance to the Principles of the 3Rs

**DOI:** 10.3389/fvets.2019.00195

**Published:** 2019-06-19

**Authors:** Adriaan de Jong

**Affiliations:** Department of Wildlife, Fish, and Environmental Studies, Swedish University of Agricultural Sciences, Umeå, Sweden

**Keywords:** principle of the 3Rs, redundant measurements, anser fabalis, R script, welfare, bird studies

## Abstract

The Principles of the 3Rs apply to animal use in research regardless where the research is conducted. In wildlife research, particularly research on wild birds, 3R implementation lags behind research using laboratory, farm, or pet animals. Raised 3R awareness and more field-adapted techniques and protocols are expected to improve the situation. Unpredictable access to animals entices the wildlife researcher to make the most of each caught animal, leading to potential over-use, and violation of the 3Rs. In this study, I statistically screened an existing set of Bean Goose biometric data for the presence of redundant measurements. The results show that it was possible to distinguish between the *fabalis* and *rossicus* subspecies (the original aim of the measurements) with fewer measurements (2 vs. 17). Avoidance of the redundant measurements was estimated to reduce both handling time and welfare impact with c. 80%. A robust scheme, supported by an R-script, is presented for continuously weeding out redundant measurements. This scheme is potentially applicable for measurement protocols in any wildlife study, and thus, contributes to the implementation of the principals of the 3Rs in wildlife research in general.

## Introduction

Unlike in research using laboratory and farm/domestic animals, access to animals in wildlife research is often highly unpredictable. No matter how skillful and well-informed the staff is, it takes favorable circumstances and a stroke of good luck to e.g., dart a moose or catch a migrant bird. Once the animal is finally available, the researcher is tempted to make utmost use of the occasion, and thus, measure and sample “as much as possible.” When challenged, the use of the individual animal beyond the core purpose of the study could easily be motivated with e.g., data sharing and bio-banking in an *a posteriori* manner. How does such opportunistic sampling behavior match the legal and moral requirements of the use of animals in research?

The Principles of the 3Rs are at the core of modern regulations of the use of animals in research and education (1–3). Originally formulated by Russell and Burch (4), the Principles of the 3Rs (“the 3Rs”) prescribe a continuous process aiming to *Replace* live animals with other study systems (e.g., cell cultures or computer models), to *Reduce* the numbers of animals used without jeopardizing the quality of the research and, finally, *Refine* the conditions for the individual animals truly needed for the experiment. The 3Rs apply independently from legal definitions of “animals used in experiments and teaching” and thus, the requirements for approval by an Ethical Committee on Animal Experiments (“ECAE”). In addition to positive effects on animal welfare, the implementation of the 3Rs is known to improve research quality through better planning and the development of novel methods and practices [e.g., (5)].

The 3Rs are now firmly rooted and routinely implemented in laboratory research (6, 7). Also in research based on the use of farm and domestic animals, the 3Rs are rapidly gaining momentum (8). In both fields, a predictable research environment facilitates a strict application of methods and protocols, high-quality care taking and housing, and properly organized and educated staff. The main body of the EU and Swedish national regulations for animals used in research were developed for these research environments, but they also apply to wildlife research (2, 9). In their review of the implementation of the 3Rs in wildlife research, (10) sorted out the challenges and possibilities for bringing wildlife research in par with practices in the laboratory and the farm. They concluded that raised 3R-awareness and field-adapted methods and protocols were important factors for successful implementation.

In most wildlife research, the animals are the object of study rather than a means to study other phenomena (e.g., toxicity or medical treatment). For this reason, the wildlife researcher has an inherently genuine interest in the well-being and functioning of the included individuals. How well this interest is materialized depends on the species-specific veterinary knowledge and skills of the research team, as well as the organization and the toolbox of the operation. In ornithology, unpaid volunteers and amateur researchers do most of the fieldwork (11–13), and their competence in the field of animal welfare, the 3Rs and research planning is often insufficient.

The numbers of wild birds subjected to scientific experimentation are unknown. For e.g., Sweden, which has a special definition of animals used in scientific experimentation (14), the official statistics for animals used in research (15) do not separate numbers for different research environments. Together with colleagues, I have estimated the number of wild birds used in research in Sweden to be c. 10,000 annually. To this number, c. 300,000 birds subject to “normal” ringing should be added (16). Bird ringing does not require ECAE approval in Sweden and most other countries.

Bird ringing not only involves catching and putting a metal ring around the leg of a bird, but also collecting data on weight, wing length, molt patterns, fat scores, etc. These all add to the overall time the bird is held captive and the level of human-induced stress the individual bird is exposed to. Handling times and levels of invasiveness are assumed to be valid proxies for negative impacts on welfare and fitness [e.g., (17–19)]. Consequently, a reduction of handling time and/or avoidance of particularly invasive treatments would improve the well-being of the wild birds used in research. From a 3R point of view, the fringe benefit of each additional measurement or treatment must be shown to out-weigh the negative effects.

Large avian herbivores (e.g., swans, geese, and cranes) wintering in the agricultural landscapes of temperate Eurasia and North America have increased dramatically in numbers over the last decades (20, 21). The Bean Goose *Anser fabalis* is one of the few exceptions to this general trend, with a stable population at best (21, 22). The Bean Goose has a complex and long-debated population structure (23–28) and several subspecies and sub-populations are in marked decline (29, 30). Throughout its range, the Bean Goose is subject to both regulated and illegal hunting and, when in conflict with agricultural interests, protective shooting, and scaring (14, 31–33). For successful international management and conservation of all relevant components of the Bean Goose population there is a great need of discrimination criteria and range delineation data (30, 34). Various on-going research projects try to provide this information [e.g., (35–37)]. The data set used in this study was generated as part of this endeavor.

I will explore the presence of redundant measurements in this existing set of Bean Geese biometrics data. The outcomes of the statistical analyses will then be discussed in the light of animal welfare and the implementation of the Principles of the 3Rs. From this, I will conclude on a 3R-adapted strategy for the development of measuring and sampling protocols for research with unpredictable access to wild birds. Because non-academics play an important role in ornithological research, this strategy will be designed to fit even this category of researchers.

## Materials and Methods

### The Dataset

The existing measurement dataset had been collected by Dipl.Biol. Thomas Heinicke, Germany, from live geese during various catching operations in Germany, Finland, Norway and Sweden 2007–2012 (Table 1, full data set as Supplementary Material). These independent operations each had a full range of relevant permissions, including animal research ethics approval. The core aim of the data collection was the discrimination between Taiga and Tundra Bean Geese (*Anser fabalis fabalis* and *A. f. rossicus*, respectively). Based on the expert knowledge of Mr. Heinicke, the geese were classified as either *fabalis* or *rossicus* from a combination of body structure (habitus), location, and season. The measurements were intended to be descriptive at first, but decisive when used in future goose studies (38, 39). The measurements were part of protocols that, depending on the catching operation at hand, also included e.g., weighing, aging, sexing, marking, and DNA sampling. For the sake of this study, the measurement data were taken “as are” and without scrutiny of measuring technique and instrumental error. In addition, the subspecies classifications (182 *rossicus* and 80 *fabalis*) were taken as ground truth. All measurements were made with a mechanical caliper to the nearest 0.1 mm, except the tail length, which was measured with a ruler to the nearest multiple of 0.5 mm [c.f. (40)]. Numbers of tomia (further referred to as “Teeth” in accordance to common vocabulary among field-ornithologists), were determined by visual inspection.

**Table 1:** Origin of the sampled individual.

**Subspecies**	**Finland**	**Germany**	**Norway**	**Sweden**	**Sum**
Fabalis	1	15	1	63	80
rossicus	–	118	38	26	182
Sum	1	133	39	89	262

The dataset contains 17 potentially explanatory variables (Table 2) and one response variable (*fabalis* = 1, *rossicus* = 0). Variable names are given in brackets in the text. For improved readability, the full variable names were replaced by single letter names (A-Q) in the output of some analyses (e.g., correlation matrix). The creation of new (composite) variables from existing ones (e.g., “Bill shape” = “Bill height” / “Culmen”) may seem appealing, but composite variables require special statistical considerations and thus, were largely avoided. The only exception was “Nail shape” = “Nail length” / “Nail width,” because the shape of the nail (clypea) on the bill was considered to be a strongly discriminating feature and recorded separately as a categorical variable (and thus unsuitable for most analyses used here). Potentially, this dataset allows for a huge amount of combinations of existing variables, with and without interactions. Because the aim of this study was the reduction of measurements and thus variables, rather than finding the best models, I chose to include only a few variable combinations at the final stages of model selection.

**Table 2:** Full and abbreviated variable names and a short description of their biological meaning.

**Variable**	**Abbreviated**	**Description**
Culmen	A	Distance from tip of bill to forehead
Lower mandible	B	Length of the lower mandible
Bill tip to nostril	C	Distance between the tip of the bill and the nostril
Bill plus head	D	Length of bill and head
Head length	E	Length of head from the base of the bill
Head width	F	Width of head across the “cheeks”
Nail length	G	Length of the nail on the bill
Nail width	H	Width of the nail on the bill
Bill height	I	Height of the bill at its base
Bill height nail	J	Height of the bill right behind the nail
Bill height nostril	K	Height of the bill right in front of the nostril
Bill width	L	Width of the bill at its base
Height lower mandible	M	Maximum height of the lower mandible
Tail	N	Length of tail
Tarsus	O	Length of the tail
Toe nail	P	Length of the tarsus
Teeth	Q	Number of teeth in the upper mandible (one side)

### Statistical Analysis

I screened the full set of potentially explanatory variables for subspecies determination by stepping through a number of statistical analyses on single, pairwise, and multiple variables (R functions in brackets, R script in Supplementary Material).

#### Individual Variables

After listing the basic statistics for individual variables, I visually inspected their frequency distributions (“histogram”) and incidence plots of their logistic models for subspecies discrimination (“glm, family = binomial”).

#### Pairwise Variables

First I plotted the observations against all variable pairs (“pairs”) and the correlation matrix (“cor” and “corrplot”). To exemplify the effect of the number of measured individuals, I also produced the correlation matrix analysis on a small (*N* = 20) random sub-set of the data. I then used partitioning of the observations on pairs of variables (“kmeans,” a simple form of cluster analysis) to visualize how well variable pairs could distinguish the subspecies.

#### Multiple Variables

I used discriminant analysis (“lda”) and AICc-based model selection of the logistic models (“aictab”) for multiple variable analyses. In the latter, I also included a selection of composite and multi variable models. Based on the results of the model selection process, I checked the quality of discriminant models for strongly reduced numbers of potentially explanatory variables (*n* = 5 and *n* = 2). Although Principle Component Analyses (PCAs) are popular for multivariate analyses and can produce visually appealing output, I chose to avoid PCA because they require pre-treatment of the input variables and their output is difficult to interpret [e.g., (41)]. In addition, PCAs aim to conserve rather than challenge existing variables, and thus, are less suitable for the purpose of this study.

All statistical analyses were made in R 3.4.4 x64 (42), with additional packages AICcmodavg (43), corrplot (44), lattice (45), lme4 (46), MASS (47), and Matrix (48), and supporting packages these depend on.

### Animal Welfare

Based on personal experience from participating in most of the catching operations behind the dataset, I took times for taking the various measurements on a mock-up goose. I also estimated the times used for additional procedures of the most extensive protocol (**Table 8**). Times for catching and storage (in bags) were not included, because these vary dramatically with circumstances; many of which are beyond the control of the research team. All estimates assume the team to be well-trained and well-equipped for outdoor conditions. Estimates also assume that a dedicated staff member takes notes and other members take care of the logistics (e.g., photo-documentation and releasing the birds). Consequently, all estimates of handling times are conservative. In addition to handling times, I subjectively scored the level of invasiveness of each procedure on scale 1–10, with 10 being the highest level.

## Results

**Figure 1:**
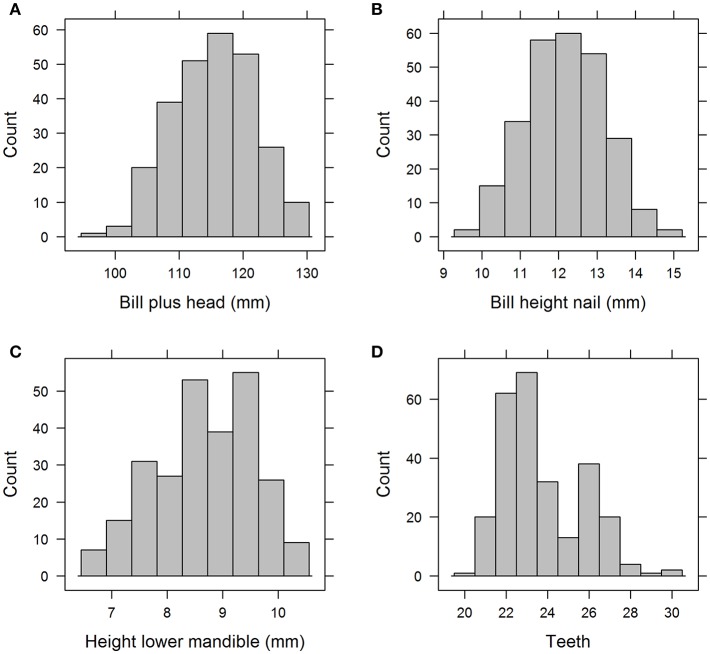
Histograms of a selection of the 17 variables, “Bill plus head” **(A)** and “Bill height nail” **(B)** with unimodal distribution vs “Height lower mandible” **(C)** and “Teeth” **(D)** with bimodal distribution.

The basic statistics (minimum, maximum, range, mean, standard deviation, and median) of all explanatory variables are presented in Table 3. The frequency distribution of a selection of four variables are shown in Figure 1. The upper two histograms (“Bill plus head” and “Bill height”) show unimodal distributions indicative of normal distribution across the entire sample. The lower two (“Height lower mandible” and “Teeth”) show bimodal distributions indicative of sub-grouping of the individuals based on these characteristics.

**Table 3:** Basic statistics.

**Variable**	**Min**	**Max**	**Range**	**Mean**	**SD**	**Median**
Culmen	45.2	67.2	22.0	57.5	4.4	57.7
Lower mandible	42.9	65.9	23.0	55.3	3.6	55.9
Bill tip to nostril	25.1	39.1	14.0	30.5	2.0	30.6
Bill plus head	96.0	129.0	33.0	115.4	6.4	116.0
Head length	57.8	72.8	15.0	65.5	2.8	65.4
Head width	26.7	43.5	16.8	38.0	2.1	38.2
Nail length	12.5	21.3	8.8	16.1	1.3	16.1
Nail width	10.0	16.3	6.3	13.7	0.9	13.8
Bill height	27.0	35.4	8.4	30.8	1.6	30.9
Bill height nail	9.5	15.0	5.5	12.2	1.0	12.2
Bill height nostril	14.0	21.7	7.7	18.0	1.5	18.1
Bill width	20.2	29.8	9.6	26.6	1.3	26.7
Height lower mandible	6.6	10.4	3.8	8.7	0.9	8.7
Tail	90.0	152.5	62.5	123.3	11.3	124.8
Tarsus	60.4	89.1	28.7	77.1	4.9	77.1
Toe nail	64.9	104.0	39.1	86.2	6.9	86.6
Teeth (N)	20	30	10	23.7	−	23.0

*All measurements in mm except the number of “Teeth” on the upper mandible (integer). N = 262 for all variables. Min = minimum value, Max = maximum value, Range = Max − Min, SD = standard deviation*.

**Figure 2:**
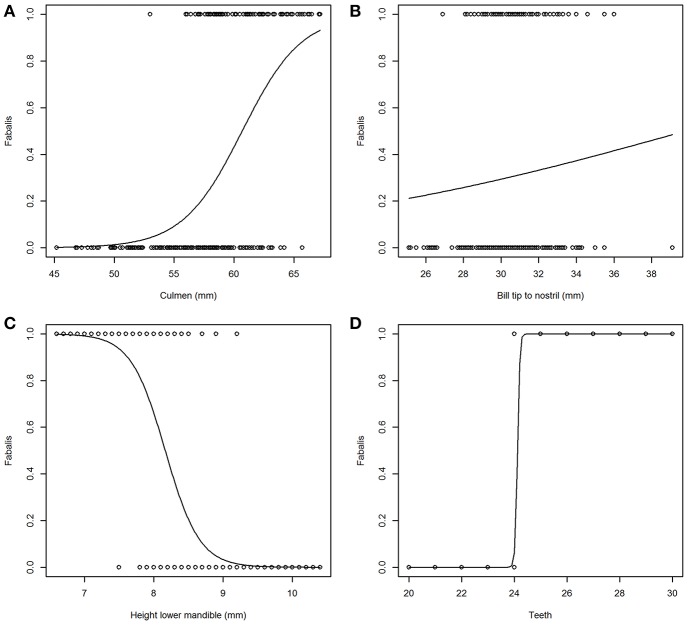
Incidence plots from a selection of logistic models for subspecies (*rossicus* = 0, *fabalis* = 1). Predicted responses go from poor in “Bill tip to nostril” **(B)** to distinct in “Teeth” **(D)**. Variables “Culmen” **(A)** and “Height lower nmandible” **(C)** show intermediate, contra-directional responses.

Incidence curves for logistic models based on four individual variables are presented in Figure 2. Figure 2A shows a clear but not abrupt relationship between “Culmen” and subspecies (*fabalis* birds have longer culmen) in contrast to Figure 2B with virtually no effect of “Bill tip to nostril” (in *fabalis* and *rossicus* birds the distances are very similar). For “Height lower mandible” (Figure 2C), the curve dips fairly steep indicating a firm strong relationship with subspecies (*rossicus* birds have greater height = more pronounced “grin”). The variable “Teeth” (Figure 2D) reveals a very strong relationship with subspecies expressed as a sharp break at 24 teeth (*fabalis* birds have more teeth than *rossicus*). The model based on “Teeth” had by far the lowest AICc value (19) and thus, fitted the data best (Table 4). The “Height lower mandible” model came second (AICc = 136) while all other models had AICc > 189.

**Table 4:** AICc scores for all 17 single variable logistic models.

**Variable**	**AICc**
Culmen	235.4
Lower mandible	268.1
Bill tip to nostril	324.6
Bill plus head	245.8
Head length	315.7
Head width	324.5
Nail length	274.6
Nail width	295.4
Bill height	325.7
Bill height nail	303.8
Bill height nostril	207.3
Bill width	313.4
Height lower mandible	135.6
Tail	224.9
Tarsus	211.7
Toe nail	189.7
Teeth	19.0

*The lower the AICc, the better the model fitted the data*.

**Figure 3:**
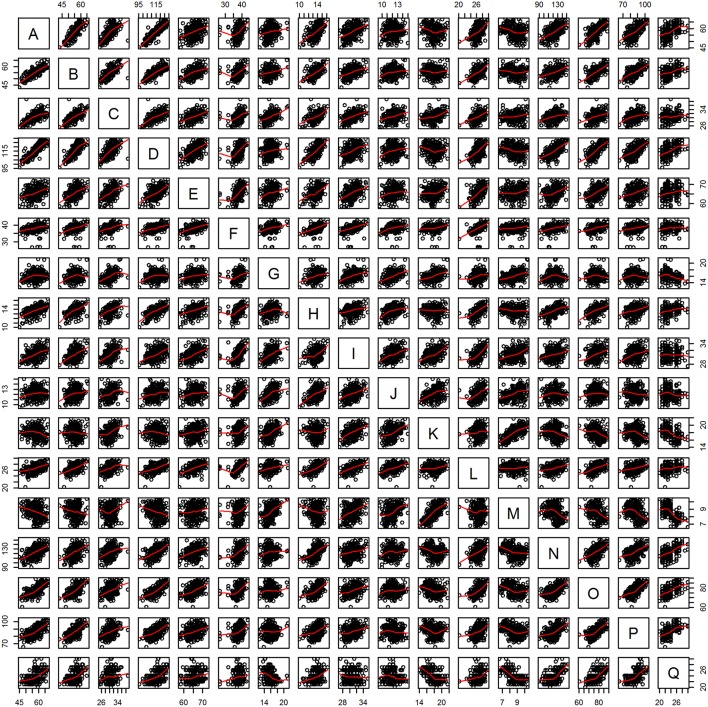
Panel plot of observations against pairs of variables. Trend lines in red. See Table 2 for variable name acronyms.

The Pairs plot (Figure 3) shows that observations are either aggregated along a trend line (indicative of correlation) or seemingly randomly dispersed across the plot area (indicative of absence of distinct grouping). In a plot with this many variables, the details of the distribution are not visible, though. For more detailed analysis, separate plots of variable pairs (“plot(x, y)”) are suggested (not included here, but described in the R scripts in Supplementary Material).

**Figure 4:**
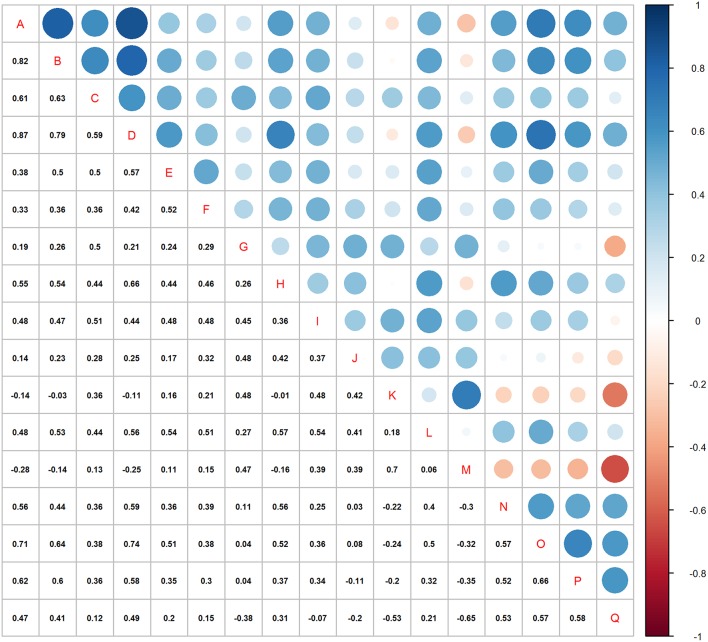
Correlation matrix for all 17 variables in the analyses. See Table 2 for variable name acronyms.

The correlation matrix (Figure 4) shows strong correlation between many of the variable pairs (high correlation coefficients and large dots). Most (85%) of the correlations were positive and 3.7% had >0.7 coefficients (8.1% > 0.6).

**Figure 5:**
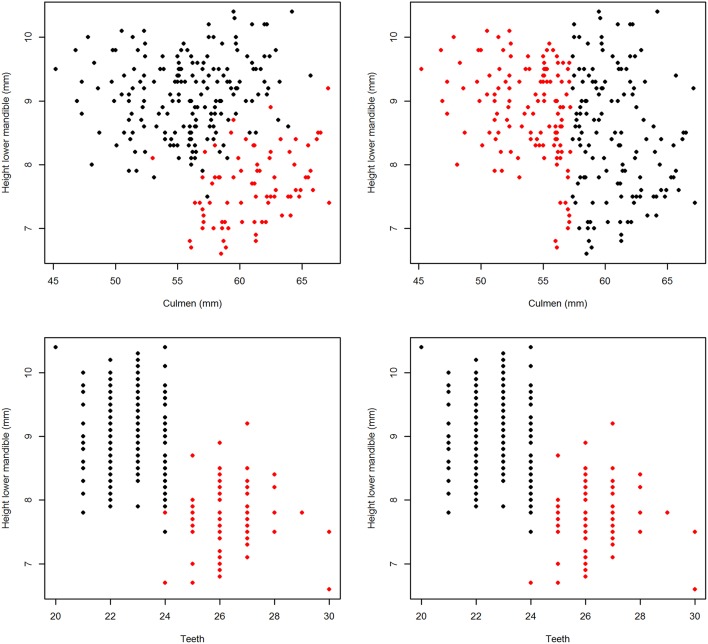
Plots of individual Bean Geese (*fabalis* = red, *rossicus* = black) on pairs of variables. (Left) original subspecies classification (ground truth). (Right) classification by the “kmeans” function.

Two pairs of plots of the results of partitioning are presented here (Figure 5). For combination of “Culmen” and “Height lower mandible” the real and modeled distribution of the two subspecies are clearly different (upper panels). For the combination of “Teeth” and “Height lower mandible” the patterns of real and modeled distributions are almost identical (lower panels). The kmeans model based on the first pair of variables assigned only 30.5% of the individuals correctly while the second was accurate in 99.6% of the cases (Table 5).

**Table 5:** Assignment of individuals to subspecies class by the kmeans models against the ground truth classification.

	**Fabalis—ground truth**	
**A**		
Fabalis–model	0	1
0	75	75
1	107	5
**B**		
Fabalis—model	0	1
0	182	1
1	0	79

*A = scores by model based on “Culmen” and “Height lower mandible”, B = scores by model based on “Teeth” and “Height lower mandible”*.

**Figure 6:**
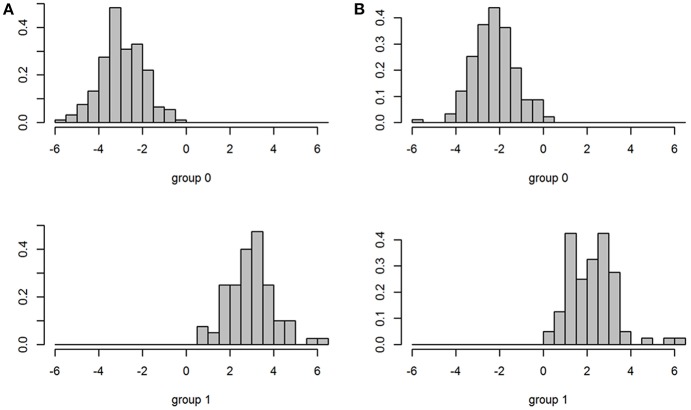
Plots of discrimination between subspecies (Group 0 = *rossicus* and Group 1 = *fabalis*) for models based on all 17 variables **(A)** and only two variables **(B)**.

The *fabalis* and *rossicus* subspecies were well separated by the discriminant model based on all variables (Figure 6A). The linear discriminant coefficients (LDs) were highest for “Height lower mandible,” “Teeth” and “Nail length” (LD1 = −0.76, 0.62, and −0.36, respectively). Twelve (70%) of the variables had coefficients <0.1 and thus, contributed little to the discrimination process (Table 6). After removing all LD1 <0.1 variables, the remaining five variables still separated the subspecies nicely. Even with only “Height lower mandible” and “Teeth” included, the overlap between the subspecies was very small (Figure 6B). In the latter model, the coefficients were equally strong, but of opposite sign (−0.88 and 0.88, respectively).

**Table 6:** Linear discriminant coefficients for model based on all 17 **(A)**, five **(B)** and two variables **(C)**.

	**LD1**
**A**	
Height_lower_mandible	−0.765
Teeth	0.624
Nail_length	−0.356
Bill_height_nostril	−0.249
Bill_width	0.120
Bill_height	0.098
Bill_height_nail	−0.093
Head_width	−0.086
Lower_mandible	0.067
Head_length	0.064
Toe_nail	0.062
Culmen	0.057
Bill_tip_to_nostril	−0.054
Tail	0.031
Bill_plus_head	−0.023
Nail_width	0.020
Tarsus	0.013
**B**	
Teeth	0.805
Height_lower_mandible	−0.753
Bill_width	0.303
Bill_height_nostril	−0.239
Nail_length	−0.101
**C**	
Teeth	0.880
Height_lower_mandible	−0.878

In the formal AICc-based model selection process for the single variable logistic models (Table 7A), the “Teeth” variable virtually absorbed the entire AICcweight and thus, left very little credit for the other models. After adding four logistic models based on one composite variable (“Nail shape”) and three variable combinations, “Teeth & Height lower mandible” and “Teeth & Nail shape” proved to fit the data better than the “Teeth” variable alone (Table 7B). The difference between the top three models and the next was large (ΔAICc > 116).

**Table 7:** AICc-based model selection for the 17 original single variable models **(A)** and the extended variable set **(B)**.

	***K***	**AICc**	**ΔAICc**	**AICcWt**	**Cum.Wt**	**LL**
**A**						
Teeth	2	19.0	0.0	1	1	−7.5
Height lower mandible	2	135.6	116.6	0	1	−65.8
Toe nail	2	189.7	170.7	0	1	−92.8
Bill height nostril	2	207.3	188.3	0	1	−101.6
Tarsus	2	211.7	192.7	0	1	−103.8
Tail	2	224.9	205.9	0	1	−110.4
Culmen	2	235.4	216.4	0	1	−115.7
Bill plus head	2	245.8	226.8	0	1	−120.9
Lower mandible	2	268.1	249.1	0	1	−132.0
Nail length	2	274.6	255.6	0	1	−135.3
Nail width	2	295.4	276.4	0	1	−145.7
Bill height nail	2	303.8	284.8	0	1	−149.9
Bill width	2	313.4	294.4	0	1	−154.7
Head length	2	315.7	296.7	0	1	−155.8
Head width	2	324.5	305.5	0	1	−160.2
Bill tip to nostril	2	324.6	305.6	0	1	−160.3
Bill height	2	325.7	306.7	0	1	−160.8
**B**						
Teeth & Height lower mandible	3	11.7	0.0	0.89	0.89	−2.8
Teeth & Nail shape	3	16.4	4.7	0.09	0.98	−5.1
Teeth	2	19.0	7.3	0.02	1.00	−7.5
Height lower mandible	2	135.6	123.9	0.00	1.00	−65.8
Nail shape	2	182.4	170.7	0.00	1.00	−89.2
Culmen & Bill height	3	186.7	175.0	0.00	1.00	−90.3
Toe nail	2	189.7	178.0	0.00	1.00	−92.8
Bill height nostril	2	207.3	195.6	0.00	1.00	−101.6
Tarsus	2	211.7	200.0	0.00	1.00	−103.8
Tail	2	224.9	213.2	0.00	1.00	−110.4
Culmen	2	235.4	223.7	0.00	1.00	−115.7
Bill plus head	2	245.8	234.1	0.00	1.00	−120.9
Lower mandible	2	268.1	256.4	0.00	1.00	−132.0
Nail length	2	274.6	262.9	0.00	1.00	−135.3
Nail width	2	295.4	283.8	0.00	1.00	−145.7
Bill height nail	2	303.8	292.1	0.00	1.00	−149.9
Bill width	2	313.4	301.7	0.00	1.00	−154.7
Head length	2	315.7	304.0	0.00	1.00	−155.8
Head width	2	324.5	312.8	0.00	1.00	−160.2
Bill tip to nostril	2	324.6	312.9	0.00	1.00	−160.3
Bill height	2	325.7	314.0	0.00	1.00	−160.8

*K = number of model parameters. AICc = , ΔAICc = difference in AICc's between the best and the current model, AICcWt = AICc weight, Cum.Wt = accumulated AICcWt, LL = loglikelihood value*.

The original 17 measurements took an estimated 209 s (3.5 min) to perform (Table 8). Based on the statistical analyses in this study, the number of measurements could have been reduced to only two (“Height lower mandible” and “Teeth”) without significant loss of discriminating power in subspecies identification. This reduction would have brought down the estimated time needed to take the necessary measurements to 37 s, 18% of the original time (Table 8).

**Table 8:** Estimated duration (seconds) for actions in this study and in protocols for Bean Goose catching operations, with potential reductions based on the results of this study.

			**Times (seconds)**			
					**Protocol**	
**Procedure**	**Type**	**Invasive**	**Study**	**Reduced**	**Full**	**Advised**
Startup	Handling	4			30	30
Ringing	Handling	6			60	60
Bill color (% orange)	Visual inspection	1			5	5
Bill shape-color code	Visual inspection	1			8	
Shape of nail (round/oval)	Visual inspection	1			5	5
Shape of the nostril	Visual inspection	1			5	
Bill plus head	Measurement	4	15		15	15
Head length	Measurement	3	12		12	
Head width	Measurement	3	12		12	
Bill width	Measurement	3	10		10	
Culmen	Measurement	3	7		7	
Bill tip to nostril	Measurement	3	8		8	
Nail length	Measurement	3	7		7	
Nail width	Measurement	3	7		7	
Length lower mandible	Measurement	3	10		10	
Bill height	Measurement	3	8		8	
Bill height nostril	Measurement	3	9		9	
Bill height nail	Measurement	3	9		9	
Height lower mandible	Measurement	4	12	12	12	12
Teeth	Count	5	25	25	25	25
Tarsus	Measurement	4	30		30	
Toe nail	Measurement	3	8		8	
Wing length (flat wing)	Measurement	6			25	
Tail	Measurement	5	20		20	
Length bill – sternum	Measurement	6			30	
Total length	Measurement	7			80	
Photos of the head	Handling	2			25	25
Aging	Visual inspection	4			5	5
Sexing	Visual inspection	10			120	
Feather sampling	Sampling	8			20	20
Finishing	Handling	4			20	20
		Total	209	37	647	222
		Percent	100	18	100	34

*Invasive = subjective invasiveness score of the procedure*.

Across the full protocol of Bean Goose catching, overall time for handling an individual bird was estimated to 647 s (10.8 min). Based on the results of this study and the use of genetic sex markers, the completion of the protocol could be reduced by an estimated 66% (Table 8).

## Discussion

In this dataset, two variables proved sufficient to distinguish between the two subspecies, the core objective of the data collection. The other 15 variables contributed virtually nothing and thus, should be considered redundant in this context. If these had been omitted from the measurement protocols, the 262 Bean Geese behind this study would have experienced an estimated reduction of 82% in time. Novel research is needed to reliably quantify the welfare impact of reduced measurement protocols, but the invasiveness scores of individual measurements (Table 8) suggest that some reductions are likely to have a greater impact than others.

The role for subspecies identification of the number of tomia (“teeth”) in the upper mandible and of the maximum height of the lower mandible (referred to as “grin” by field ornithologists) were commonly known before the sampling started [e.g., (49)]. The other variables were either proxies for size (*fabalis* is generally larger than *rossicus*, but so are males relative females) or indicators of complex features, e.g., “elongated bill” in *fabalis* vs. “short and distinct” bill in *rossicus*. Characterizing these shapes would often require the construction of composite variables (e.g., “Culmen”/“Bill height”). Composite variables often have complex error structures and thus, are statistically problematic (41). The perception of “jizz” (an overall, vague appearance/impression often used by birdwatchers) is difficult to frame with a simple set of measurement data. This example shows that the measurements taken failed to do so.

The dominance of “Teeth” and “Height lower mandible” was visible through the full chain of analyses. They were the only ones with a bimodal frequency distribution and showed the steepest curves in the incidence plots of the single variable logistic models (Figures 1, 2, respectively). Obviously, the use of logistic models is inappropriate for response variables with more than two classes. In these cases ANOVA or other classes of models should be used. The other components of the chain of analyses presented here would still be valid for non-binomial response variables.

Due to the high number of potentially explanatory variables, the “pairs” plot was not very informative (Figure 3), but a closer look at the plots for single pairs would have revealed more structure in the plots for the truly informative variables than the rest. The correlation matrix (Figure 4) showed that many variables were positively correlated. Strong positive correlations are indicative of redundant variables. Many of these correlated variables were associated with the size of the birds. In a PCA or Factor analysis, many of these variables had probably been bundled into a common PCA or Factor. In the light of this study, this would confirm that most of the bundled variables should have been omitted from the protocol. The use of plots and tables from “kmeans” showed that a combination of two variables could distinguish the subspecies adequately (Figure 5; Table 5).

For this dataset, the discriminant analysis separated the subspecies very well (Figure 6). The use of linear discriminant coefficients (Table 6) for the selection or omission of variables may be misleading if done in isolation (because the variables interact in the model). Here I used this technique as an integrated part of a screening scheme, which reduced most of the risks of sorting out important variables. With only two remaining variables, the subspecies separation was still good (Figure 6). In the final model selection step, the dominance of the “Teeth” variable stood out sharply among single variable models (Table 7). The effect of additional models confirmed this dominance and showed the potential of combining variables in model building. In this case, better models were constructed from the same duo of key variables and thus, did not motivate retaining other measurements. In cases when optimal models are important, more supporting variables (and thus more measurements) might be desirable, but the fringe benefit of keeping or introducing more variables needs to outweigh the negative impact on the birds exposed by the treatment.

From a statistical point of view, there are issues that could be brought up, especially if this variable screening strategy needs to be fully applicable to “problematic” datasets (e.g., datasets with diverse data quality levels or highly skewed variables), but this is beyond the scope of this study. My aim has been to demonstrate a simple, yet robust scheme for weeding out redundant variables and thus, omit unnecessary procedures in line with the Principles of the 3Rs. The supplemented R script can be used in this process.

This study was based on a single dataset of Bean Goose biometrics and further studies to demonstrate the potential of 3R implementation by reduced measuring are wanted. The levels of reduction in handling time shown in this example are highly encouraging and indicate significant 3R potential of reduced measurement protocols in wildlife research in general. Novel research is needed to reliably quantify the welfare impact of reduced measurement protocols, but the invasiveness scores of individual measurements (Table 8) suggest that some reductions are likely to have a greater impact than others. The search for redundant measurements will also raise 3R awareness in general, pointed out as a strong driver of improved animal welfare by Lindsjö et al. (10).

This study is also a good example of how existing data can be used to gain more knowledge; a case of combined *Replacement* and *Reduction* because no geese, only data, were handled for the purpose of this study. When applied in future studies on geese and other wildlife, the concluding recommendations will also lead to *Refinement*.

Similar schemes could also be developed for the *Reduction* of the numbers of geese and other animals used in wildlife research. Supplementary to the initial power analysis, the explanatory capacity of the collected data could be gradually evaluated and the inclusion of additional individuals halted when desired levels are reached.

## Recommendations

I recommend a continuous process of challenging the necessity of measurements taken in wildlife research. Based on clear objectives and good knowledge of the research field, a minimized initial measurement protocol should be chosen. Once the set of measurement data grows (e.g., after each catching event), the dataset should be checked for weak or redundant measurements. Their place in the protocol should then be challenged. Arguments like “You never know how these data can be used in the future!” or “Colleague X may want to have these data.” might be tempting to apply, but do no longer fit into the modern world of research using live animals. If these arguments are truly relevant, the related measurements should be included in the initial protocol.

I also recommend that ECAEs, when applicable, demand a step-by-step motivation of each planned measurement and the inclusion of a reduction scheme similar to the one presented here. Finally, I recommend complementary studies on the reduction of potentially redundant measurements in research on other taxonomic groups and in-depth evaluations of how and to what extent reduced measuring actually improves the well-being of animals used in wildlife research.

A summary of this study and the full recommendation to omit several commonly applied measurements will be presented in Goose Bulletin, the official bulletin of the Goose Specialist Group of Wetlands International and IUCN. When implemented by the international goose research community, the proposed measurement reduction strategy could ease the life of hundreds of Bean Geese and thousands of other wild geese caught and handled by researchers annually.

## Ethics Statement

Data had been collected by Dipl.Biol. Thomas Heinicke, Germany, during fully approved goose catching operations arranged by national research groups in Germany, Finland, Norway and Sweden 2007–2012. None of the measurements were taken for the purpose of this study, and the taking of the measurements was not subject to legal requirements of ECAE approval at the time and location of sampling.

## Author Contributions

The author confirms being the sole contributor of this work and has approved it for publication.

### Conflict of Interest Statement

The author declares that the research was conducted in the absence of any commercial or financial relationships that could be construed as a potential conflict of interest. The reviewer EMW declared a shared affiliation, though no other collaboration, with the author to the handling Editor.
